# Neurogenic Heterotopic Ossification: A Rare Complication of Guillain-Barré Syndrome Exacerbated by Ankylosing Spondylitis and HLA-B27

**DOI:** 10.7759/cureus.88374

**Published:** 2025-07-20

**Authors:** Abderrahim Lachhab, Mohamed Maroc, Yassine Benghali, Yassin Nkhili, Ahmed Amine El Oumri

**Affiliations:** 1 Physical Medicine and Rehabilitation, Faculty of Medicine, Mohammed First University, Oujda, MAR; 2 Physical Medicine and Rehabilitation, Mohammed VI University Hospital, Oujda, MAR

**Keywords:** ankylosing spondylitis (as), ectopic ossification, guillain-barré syndrome (gbs), hla-b27, neurogenic heterotopic ossification (nho)

## Abstract

We report an exceptional case of neurogenic heterotopic ossification (NHO) developing in a 28-year-old female with pre-existing human leukocyte antigen B27 (HLA-B27) positive ankylosing spondylitis (AS), following a severe course of Guillain-Barré syndrome (GBS). Seven days postpartum, she developed acute GBS requiring three months of intensive care, intubation, mechanical ventilation, and plasmapheresis, leading to flaccid tetraplegia and prolonged immobilization. Four months post-GBS onset, she presented with bilateral hip pain and severely restricted range of motion. Radiographs and CT scans confirmed extensive heterotopic ossification around both femoral heads. Biological workup revealed significant elevations in alkaline phosphatase (ALP), C-reactive protein (CRP), and erythrocyte sedimentation rate (ESR), alongside hypercalcemia.

This case uniquely highlights the rare occurrence of NHO after GBS (reported in only 6% of GBS patients), compounded by a potential genetic predisposition from HLA-B27-positive AS. We discuss the intricate interplay of neuroinflammation, prolonged immobility, and genetic factors in NHO pathogenesis, emphasizing the diagnostic challenges and the need for a multidisciplinary approach in such complex presentations. This report underscores the importance of considering NHO in GBS patients, especially those with pre-existing inflammatory conditions, and contributes to understanding the broader risk factors for this debilitating complication.

## Introduction

Heterotopic ossification (HO) is the abnormal formation of mature bone in soft tissues outside the skeleton [1]. This condition is categorized by its origin: neurogenic (often following brain or spinal cord injuries (SCIs)), traumatic, or genetic (such as fibrodysplasia ossificans progressiva) [1]. Neurogenic heterotopic ossification (NHO) can also be a rare complication of Guillain-Barré syndrome (GBS). For example, a study by Zelig et al. found that NHO was observed in only four out of 65 GBS patients (6%) over three years [2].

Several clinical factors influence NHO development, including the severity of central nervous system injury, spasticity, pressure ulcers, systemic infections, and prolonged immobilization. This ectopic bone formation typically begins within three months of the neurological injury, with its peak incidence often noted during the second month [1]. Heterotopic ossification commonly develops around major joints such as the hip, knee, and shoulder, frequently leading to pain and restricted joint mobility [3]. Historically, NHO has been diagnosed using imaging techniques such as plain radiographs, bone scintigraphy, CT scans, and MRIs. In its initial stages, bone scintigraphy, which shows increased radionuclide uptake, is considered the gold standard for early NHO detection [1]. More recently, ultrasonography has been proposed as a potentially safer and more cost-effective alternative for identifying NHO early on [4].

The underlying mechanisms of NHO are not yet fully understood. However, a strong connection between nervous tissue injury and soft tissue inflammation is widely recognized as crucial for its development. Recent research has highlighted the involvement of immune cells, particularly phagocytic macrophages, in the pathogenesis of NHO [1]. Furthermore, some studies suggest an association between ankylosing spondylitis (AS) and NHO, particularly in human leukocyte antigen B27 (HLA-B27)-positive patients [5].

In recent years, while surgical removal of mature NHO remains a treatment option, more effective strategies are emerging. These advanced approaches focus on understanding the underlying molecular mechanisms of HO formation. By targeting these processes, treatments aim to inhibit the pathological development of HO at various stages [6].

In this case report, we present the exceptional case of a 28-year-old female with a known history of AS, who developed GBS, complicated by the emergence of NHO approximately four months into her intensive care unit admission. This case highlights a rare confluence of these conditions, potentially suggesting the influence of a predisposing background (HLA-B27 positivity related to AS) on the development of NHO following GBS. Our objective is to emphasize the rarity of this clinical presentation and to contribute to a better understanding of the risk factors and mechanisms involved in this complex type of NHO.

## Case presentation

**Figure 1:**
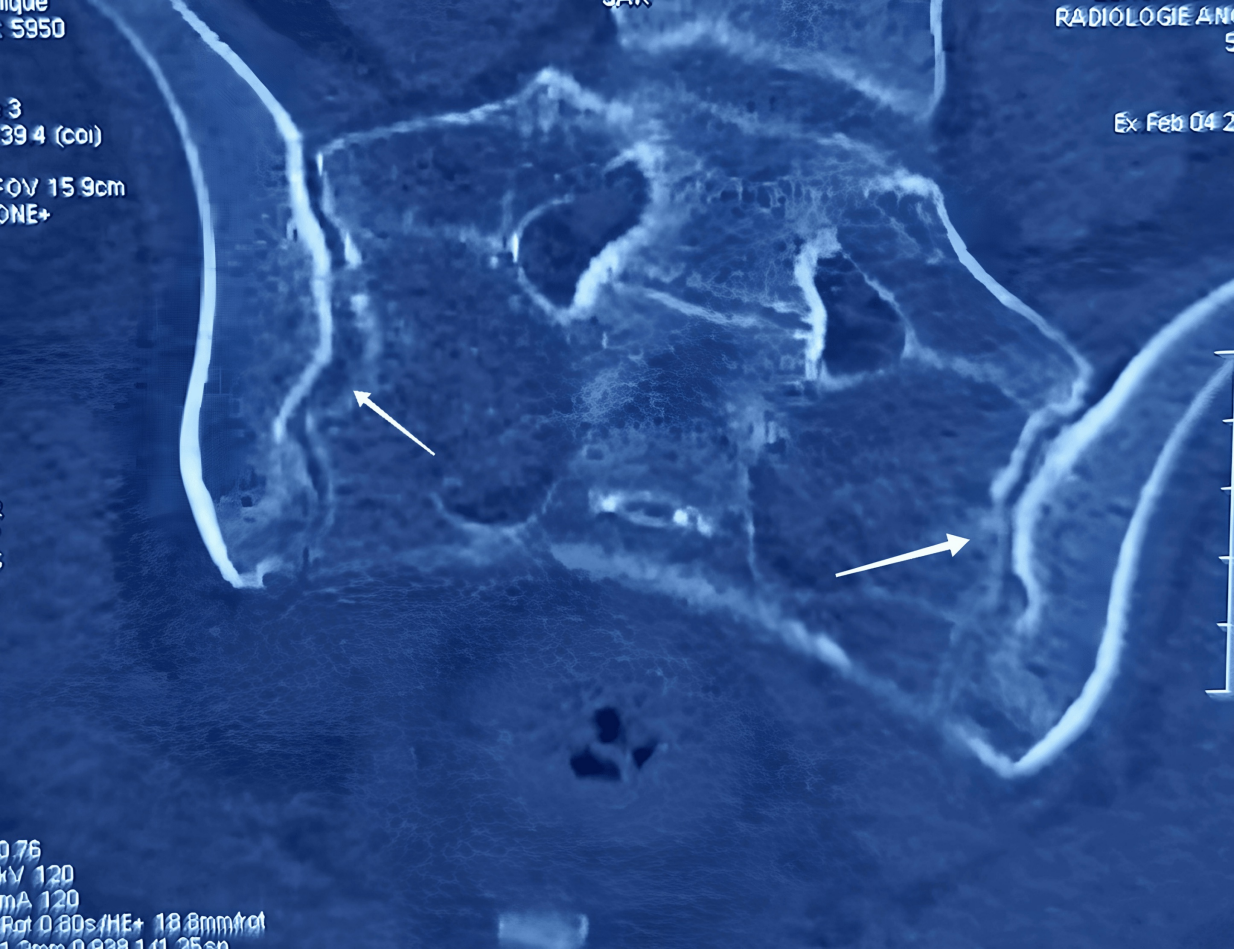
Axial CT scan of the pelvis, showing bilateral sacroiliitis, which contributed to the diagnosis of AS. Arrows indicate erosions and sclerosis of the sacroiliac joints. AS: Ankylosing spondylitis

We present the case of a 28-year-old married female, a nurse by profession, who initially presented with inflammatory low back and bilateral buttock pain, exhibiting a characteristic inflammatory rhythm. An etiological workup, guided by new diagnostic criteria, led to a diagnosis of AS. This was supported by imaging studies, including a CT scan (Figure 1). She had no significant past medical history or family history of similar conditions. The patient was advised to initiate treatment with non-steroidal anti-inflammatory drugs (NSAIDs) but declined therapy due to an ongoing pregnancy and was not taking any other medications at the time. Her pregnancy was well-monitored and culminated in a full-term, medicalized vaginal delivery under spinal anesthesia. However, seven days postpartum, the patient presented to the emergency department with an acute onset of paresthesia in both lower limbs and the right upper limb, accompanied by dysphagia. Her Glasgow coma scale (GCS) score was 14/15, and she exhibited flaccid tetraplegia. An initial cerebro-medullary MRI was unremarkable. The symptomatology rapidly worsened, marked by an altered conscious state and severe respiratory distress, with oxygen saturation dropping to 60% and tachypnea, necessitating immediate transfer to the ICU for intubation and mechanical ventilation.

A lumbar puncture revealed albumino-cytological dissociation. Electromyography (EMG) findings, characteristic of an acute demyelinating polyneuropathy affecting all four limbs, were consistent with GBS (Table 1). Following this acute phase, the patient underwent tracheostomy, received plasma exchange therapy, and initiated bedside rehabilitation sessions within the ICU. After a prolonged three-month stay in the ICU, the patient was transferred to the physical medicine and rehabilitation department.

**Table 1:** Initial EMG findings that were found to be typical for acute demyelinating polyneuropathy in GBS The study involved the assessment of nerve conduction parameters, including CMAP and SNAP, from nerves in all four limbs, specifically the ulnar, radial, and median nerves in the upper limbs, and the sural, peroneal, and tibial nerves in the lower limbs. EMG: Electromyography, GBS: Guillain-Barré syndrome, CMAP: Compound muscle action potentials, SNAP: Sensory nerve action potentials

Electrophysiological parameter	Observation in acute GBS
Nerve conduction velocities (motor and sensory)	Severely reduced
Distal latencies (motor and sensory)	Markedly prolonged
F-wave latencies	Significantly prolonged or absent
Compound muscle action potentials (CMAP)/sensory nerve action potentials (SNAP) amplitudes	Relatively preserved or mildly reduced (initially)
Conduction blocks and temporal dispersion	Often present
Overall pattern	Diffuse, predominantly demyelinating polyneuropathy

Four months after the onset of GBS, the patient developed hip pain, reporting a mixed inflammatory and mechanical pain in both hips. On clinical examination, she was non-ambulatory, requiring wheelchair assistance from a third party for mobility. Transfers and turning in bed were extremely difficult, and she required assistance for feeding and dressing. Standing was impossible, though she maintained a stable sitting balance, with no dysphagia or sphincter disorders noted at this point. Motor deficit was graded 3/5 in both upper limbs and 2/5 in both lower limbs. She exhibited significant limitation in hip flexion (80 degrees on the right and 90 degrees on the left) and abduction (30 degrees bilaterally). Other joints were noted to have a full range of motion.

**Figure 2:**
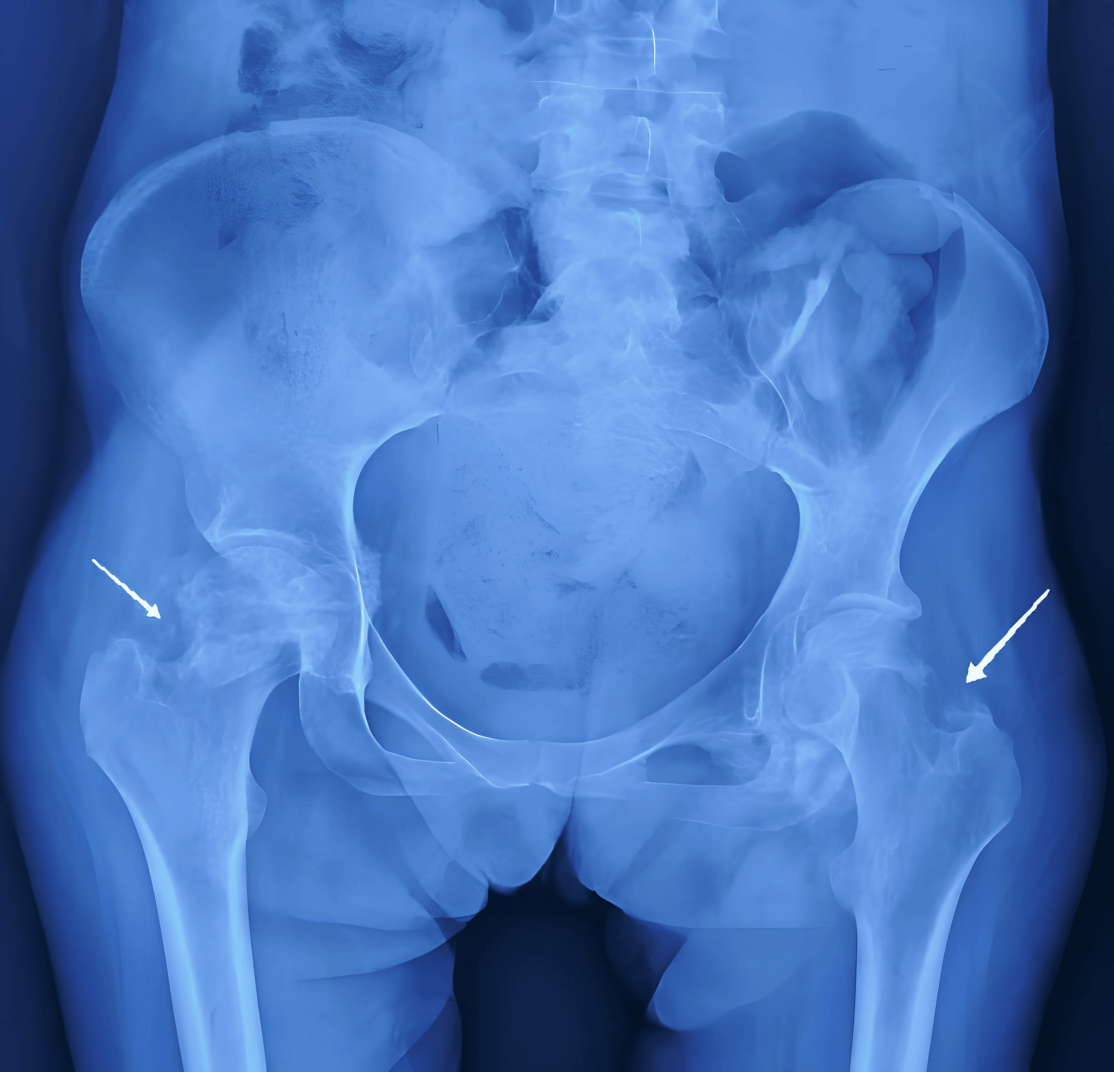
Anteroposterior radiograph of the pelvis The image reveals bilateral cloudy opacities (indicated by white arrows) around the femoral heads and necks, with relative preservation of the hip joint spaces. These findings are consistent with early HO or periarticular calcification. HO: Heterotopic ossification

**Figure 3:**
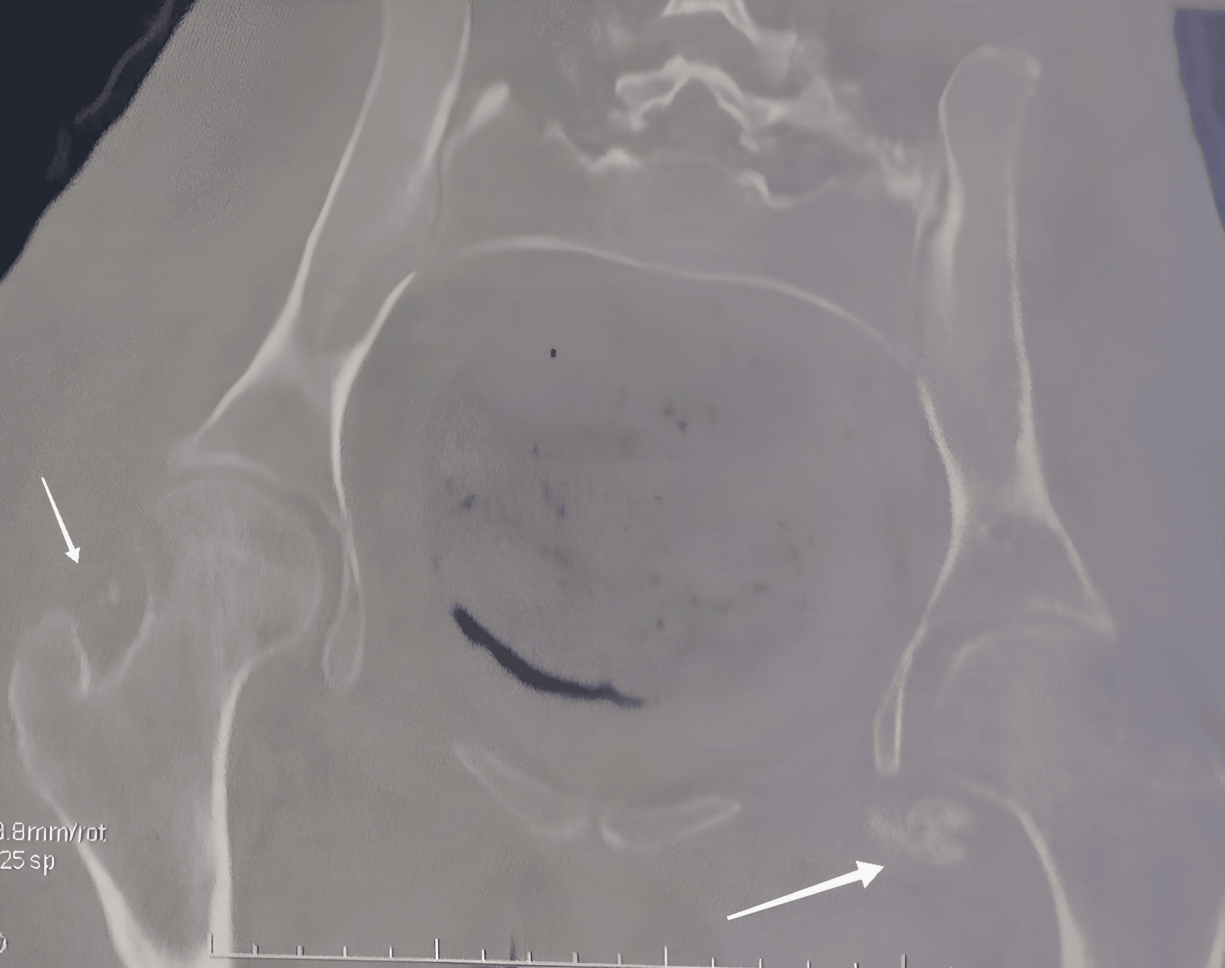
Coronal CT scan of the pelvis This image confirms the presence of bilateral HOs (indicated by white arrows) in the soft tissues around the hip joints, particularly visible anteriorly. HOs: Heterotopic ossifications

**Figure 4:**
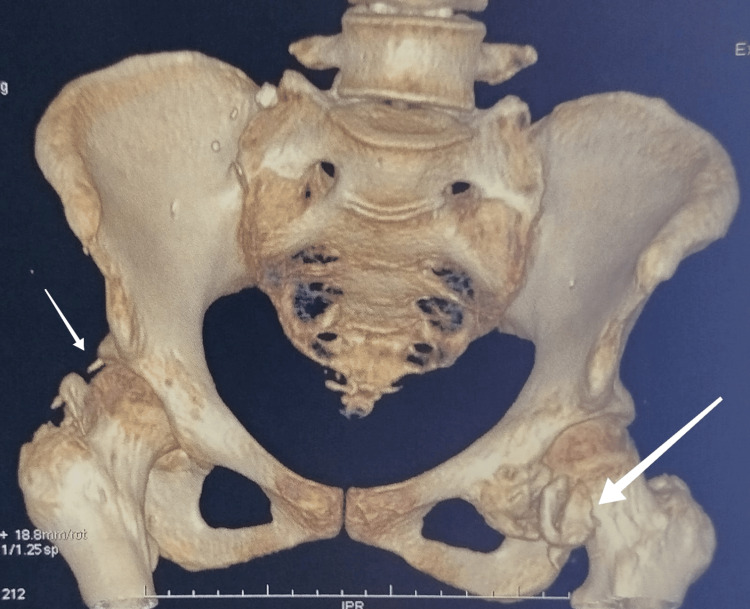
Three-dimensional (3D) reconstructed CT image of the pelvis This reconstruction provides an overview of the extensive bilateral HOs (indicated by white arrows) around both femoral necks and trochanteric regions, consistent with advanced new bone formation. HOs: Heterotopic ossifications

Initial radiographs of the hips revealed cloudy opacities around the femoral heads, with preservation of the joint space (Figure 2). A complementary injected CT scan further confirmed the presence of anterior and posterior ossifications bilaterally (Figures 3-4). Biological workup revealed hypercalcemia and hypophosphatemia with a markedly elevated alkaline phosphatase (ALP). Additionally, C-reactive protein (CRP) and erythrocyte sedimentation rate (ESR) were elevated, consistent with an inflammatory process (Table 2).

**Table 2:** Summary of quantitative laboratory findings at initial presentation along with corresponding reference ranges. ALP: Alkaline phosphatase, CRP: C-reactive protein, ESR: Erythrocyte sedimentation rate

Laboratory parameter	Initial value	Reference range	Unit	Notes
Calcium	11.2	8.6-10	mg/dL	Hypercalcemia
Phosphate	2.3	2.5 to 4.5	mg/dL	Hypophosphatemia
ALP	350	40-129	IU/L	Markedly elevated
CRP	85	< 5	mg/L	Elevated
ESR	60	<20	mm/hr	High

This case serves as a critical reminder that NHO, while rare, can be a severe complication of GBS, especially in patients with prolonged immobilization and predisposing factors. It highlights the importance of early suspicion, particularly when considering patients with concurrent inflammatory conditions like AS and relevant genetic markers such as HLA-B27. Early diagnosis and prompt, tailored management are essential to mitigate functional impairment and improve patient outcomes in these complex and challenging cases.

## Discussion

Neurogenic heterotopic ossification is the pathological formation of mature bone within soft tissues, occurring as a secondary complication to various neurological conditions [1]. While commonly observed after traumatic brain injury (TBI) or spinal cord injury (SCI) with incidences of 5% to 30% [1], NHO is a rare sequela of GBS. Zeilig et al. reported NHO in only four out of 65 GBS patients (6%) over a three-year period, underscoring its rarity [2]. Our patient's development of NHO, approximately four months after GBS onset, aligns with this rare complication.

The precise mechanisms underlying NHO are not fully understood, but a crucial link exists between nervous system damage and local soft tissue inflammation [1,6]. Current hypotheses suggest that bone morphogenetic proteins (BMPs) play a significant role. It's theorized that nerve damage, common in GBS due to demyelination, may disrupt the blood-nerve barrier (BNB), leading to neuroinflammation and elevated BMP levels [3,7]. Increased circulating BMPs, in turn, promote the differentiation of neural crest stem cells into bone-forming cells, leading to NHO [8,9]. This mechanism is particularly relevant in GBS, where the BNB is often compromised in immune-mediated neuropathies, and BMP levels are known to rise in demyelinating conditions [3,10]. Our patient's clinical course, characterized by severe GBS, prolonged immobilization, and the subsequent development of NHO, strongly supports this proposed interplay of neuroinflammation and altered bone metabolism.

Clinically, NHO typically emerges within two to four months after a neurological event, presenting with pain, swelling, redness, warmth, and reduced range of motion [1,6]. Our patient's experience mirrored these classic symptoms, with hip pain developing four months after GBS onset, accompanied by significant limitations in hip flexion and abduction. The hips are the most frequently affected site for NHO (60.9%) [7], a finding consistent with our patient's bilateral hip involvement. Zeilig et al.'s study also noted hip involvement in all GBS patients who developed NHO, with an average diagnosis at three months [2]. Other reported cases of NHO following GBS similarly feature neurological damage, extended immobility, and hip joint involvement [3,11]. Our patient's history of mechanical ventilation, incomplete tetraplegia, and prolonged immobilization further aligns with established risk factors for NHO [1,6].

Diagnostic tools for NHO include plain radiographs, bone scintigraphy, CT scans, and MRI [1]. Our patient's plain radiographs revealed characteristic cloudy opacities around the femoral heads, while a CT scan confirmed anterior and posterior ossifications bilaterally. Biologically, elevated ALP levels, typically peaking around 10 weeks, are indicative of NHO [6]. Our patient's markedly elevated ALP, hypercalcemia, hypophosphatemia, elevated CRP, and high ESR further supported the diagnosis, reflecting the active bone formation and inflammatory process. The control EMG, showing both demyelinating and axonal involvement with initial axonal recovery only in the upper limbs, underscored the severity and ongoing neurological impact of her GBS, which likely contributed to the patient's prolonged immobility and NHO development.

Beyond the known risk factors for NHO in GBS, our patient's unique presentation included a pre-existing diagnosis of AS and HLA-B27 positivity. This adds a compelling layer to her case, as research suggests a potential link between HLA typing and NHO susceptibility [12]. Specifically, studies on SCI patients have found a significantly higher occurrence of ectopic ossification in those who are HLA-B27 positive, indicating a possible genetic predisposition [5]. While further research is needed to definitively establish this link in GBS-related NHO, our case highlights the potential for such genetic factors to contribute to disease susceptibility, particularly in the context of concurrent neurological injury and systemic inflammation. This reinforces the importance of a comprehensive patient history, including genetic predispositions, when evaluating complex NHO cases.

Traditionally, NHO management has involved NSAIDs, radiation therapy, and surgical resection for mature bone [3]. Newer approaches, however, focus on molecular mechanisms. Our patient's inability to use NSAIDs due to breastfeeding led to the administration of bisphosphonates, a modern therapeutic approach that inhibits bone mineralization in early stages [6]. The multidisciplinary discussion with rheumatology and traumatology specialists will be crucial for determining optimal long-term management, including potential surgical intervention, given the functional impairment caused by the bilateral hip NHO.

This case serves as a critical reminder that NHO, while rare, can be a severe complication of GBS, especially in patients with prolonged immobilization and predisposing factors. It highlights the importance of early suspicion, particularly when considering patients with concurrent inflammatory conditions like AS and relevant genetic markers such as HLA-B27. Early diagnosis and prompt, tailored management are essential to mitigate functional impairment and improve patient outcomes in these complex and challenging cases.

## Conclusions

This case highlights the rare yet significant occurrence of NHO as a complication of GBS, particularly when compounded by pre-existing conditions like HLA-B27-positive AS. Our patient's complex presentation underscores the intricate interplay of neurological injury, prolonged immobilization, and potential genetic predisposition in NHO pathogenesis. Clinically, this case reinforces the importance of early suspicion for NHO in GBS patients, especially those with a severe course, prolonged ICU stay, or underlying inflammatory diseases. Timely diagnosis, supported by imaging and biological markers, is crucial for prompt intervention. Furthermore, the patient's unique genetic background suggests that HLA-B27 positivity might represent an additional risk factor, warranting further investigation into the genetic susceptibilities for NHO development. Ultimately, effective management of such complex cases necessitates a multidisciplinary approach, integrating expertise from neurology, physical medicine and rehabilitation, rheumatology, and orthopedics. This collaborative strategy is essential for optimizing diagnostic workup, guiding therapeutic interventions (including non-pharmacological methods and targeted medications such as bisphosphonates), and improving functional outcomes in patients facing this debilitating complication.
